# The Impact of Rural Community Elderly Care Services on the Subjective Well-Being of Older Adults: The Mediating Role of Late-Life Stress

**DOI:** 10.3390/healthcare13091029

**Published:** 2025-04-30

**Authors:** Linjing Wan, Yixin Zhu, Dan Chen, Xiuliang Dai

**Affiliations:** School of Public Policy and Administration, Xi’an Jiaotong University, No. 28 Xianning West Road, Xi’an 710061, China; wanlinjing@xjtu.edu.cn (L.W.); cd1429149424@stu.xjtu.edu.cn (D.C.); xiuliangdai@stu.xjtu.edu.cn (X.D.)

**Keywords:** China, elderly care services, subjective well-being, late-life stress

## Abstract

**Background/Objectives:** This study aims to investigate whether rural community elderly care services can enhance older adults’ subjective well-being, with a specific focus on examining whether late-life stress mediates this association. **Methods:** Subjective well-being is operationalized through three dimensions: life satisfaction, positive life attitude, and depression levels. Data were collected from a sample of 796 rural Chinese adults aged 60 years and older. Regression analysis was used to assess the direct effects of rural community elderly care services on subjective well-being outcomes, while an instrumental variable two-stage least squares model was employed to test the robustness of these findings. A mediation analysis further explored the underlying mechanisms through which these services influence well-being. **Results:** Results indicate that rural community elderly care services exert significant direct and indirect effects on all three dimensions of subjective well-being. The indirect effects arise because these services provide social support that mitigates late-life stressors and buffers the negative impacts of adverse life events, thereby enhancing psychological well-being. **Conclusions:** These findings clarify the mediating role of stress in the relationship between community care services and subjective well-being among rural older adults, highlighting the importance of addressing age-related stressors in gerontological interventions. This study contributes to the literature by providing empirical evidence for the efficacy of rural community elderly care programs and offering actionable insights for developing contextually appropriate service models to meet the needs of aging rural populations. This study elucidates how rural older adults perceive community elderly care services, providing empirical evidence for government agencies to evaluate the effectiveness of policy-driven services. It further identifies key entry points for enhancing rural care service quality and promoting elderly well-being, bridging research insights with actionable strategies for policy improvement.

## 1. Introduction

In the context of the global demographic shift, the issue of population aging has emerged as a formidable challenge confronting most countries around the world. Since 1996, both developed and developing nations have been subject to a common trend: a pronounced decline in fertility rates accompanied by a steady increase in life expectancy. This dual-trend has culminated in a dramatic upsurge in the global elderly population [1]. Notably, while developed countries are gradually transitioning into a hyper-aging state, developing countries, despite a relatively late entry into the aging phase, are experiencing a more precipitous transformation towards an aging society [2]. China, for instance, officially stepped into the ranks of aging societies in 1999. The nation’s population age structure has undergone a fundamental shift, evolving from a pattern characterized by a ‘high birth rate, low death rate, and high growth’ to one marked by a ‘low birth rate, low death rate, and low growth’. Given the long-cycle nature of population structure changes and the inherent time-lag effect, China is expected to witness a sustained expansion in older adults and an escalating degree of population aging over the long term [3]. As per the data released by the National Bureau of Statistics of China (National Bureau of Statistics website. Statistical Communiqué of the People’s Republic of China on the 2023 National Economic and Social Development [EB/OL]. https://www.stats.gov.cn/sj/zxfb/202402/t20240228_1947915.html, accessed on 1 January 2025), by the end of 2023, the number of people aged 60 and above in China approximated 297 million, with those aged 65 and above reaching around 217 million. This statistic underscores China’s position as the country with the world’s largest elderly population.

In contrast to urban regions, the aging issue in rural areas of China is far more acute. Firstly, attributable to the urban–rural divide, rural families have increasingly come to rely on migrant work in cities as their primary income source. This has led to a substantial outflow of young rural labor forces, causing a continuous rise in the proportion of older adults in rural areas. The physical separation between the younger and older generations has gradually eroded the traditional family-centered elderly care model [4]. Consequently, the care demands of older adults in rural areas have transitioned from family-based care to the pursuit of socialized elderly care services. Secondly, the relatively underdeveloped economic situation in rural areas poses significant challenges. A considerable number of elderly individuals lack a stable income stream and are mainly dependent on agricultural activities or financial support from their offspring [5]. This economic vulnerability severely restricts their options in terms of elderly care, rendering it arduous for them to afford high-cost elderly care services.

In the United States, Canada, Japan, and other countries, efforts have been made to resolve the challenges related to elderly care through the implementation of long-term care systems. However, constrained by the persistent rural service deficit, these systems have significantly impeded the rural elderly’s capacity to age in place [6]. As a result, the rural elderly are frequently compelled to relocate outside their home counties to access long-term care services. Existing research underscores that obtaining long-term care is not perceived as a voluntary ‘choice’ but rather as a forced transition [7], triggering distress among older adults due to the loss of autonomy in their lifestyle and social connections [8]. Moreover, in rural regions, the absence of a comprehensive stratified care system, combined with the relatively low socioeconomic status of residents, has effectively transformed long-term care services into a privilege primarily accessible to high-income groups, exacerbating social disparities in elderly care provision [9].

Against this backdrop, community-based elderly care services, characterized by advantages such as their proximity to home, cost-effectiveness, and high flexibility, are well positioned to offer convenient and accessible elderly care services for rural senior citizens. As a result, they have gradually emerged as a crucial approach to tackling rural elderly care issues. Firstly, the payment ability of rural elderly people in China is generally limited. According to the data released by the National Bureau of Statistics (National Bureau of Statistics website. Information on Residents’ Income and Consumption Expenditure in 2023 [EB/OL]. https://www.stats.gov.cn/sj/zxfb/202401/t20240116_1946622.html, accessed on 1 January 2025), in 2023, the per capita disposable income of urban residents reached CNY 51,821, while that of rural residents was only CNY 21,691, highlighting a substantial income gap. The cost-effective nature of the community-based elderly care service model endows it with a wider adaptability range, making it more suitable for the economic situation of rural areas. Secondly, in contrast to urban elderly people, rural elderly people, deeply influenced by the sentiment of ‘returning to one’s roots’, hold a stronger attachment to their native land. They are often reluctant to become ‘accompanying elderly’ and burden their children. Therefore, the vast majority of rural elderly people have a more pronounced preference for home-based elderly care. The rural community-based elderly care service, adhering to the principle of providing services in the vicinity, caters precisely to the practical need of older adults for ‘aging in place’. Finally, from the perspective of value identification, older adults in rural areas have a strong sense of belonging to their local communities. The community-based elderly care service model not only provides elderly care but also creates a shared space for rural elderly people to engage in social activities. Through collective participation, older adults can meet their emotional needs through interactions with their peers. In summary, rural community-based elderly care services can seamlessly integrate into the rural social structure, thereby significantly contributing to the improvement of the rural social elderly care service system [10].

Existing research has mainly demonstrated the development advantages of rural community elderly care services from two aspects: the individual elderly care service needs of rural elderly people and the development foundation of rural communities. Rural community elderly care services operate under a quasi-market supply mechanism. The government raises resources to provide infrastructure, and the community provides elderly care services to older adults, preventing older adults from giving up such services due to excessive costs [11]. In terms of operating costs, elderly care services in rural areas can be regarded as limited resources. With the increase in the number of elderly people in rural areas, the cost of their access to elderly care services will become higher and higher. Therefore, under limited conditions, the community-based elderly care model with a low-cost advantage has a broader scope of adaptability [12]. Rural community elderly care services rely on local rural villages and tailor-make and integrate elderly care service content. They integrate local elderly people into a rural community, providing endogenous power and a practical carrier for the development of rural community elderly care services. It is possible to use idle facility resources within the village to build public facilities for community elderly care services, overcoming the ‘dispersal’ of rural areas [13]. Regarding sustainable development, older adults themselves have a strong sense of belonging to rural communities. Moreover, the demand for elderly care drives older adults to form a community. Through joint practices within the group, collective emotions enable older adults to meet their emotional needs during interactions with other individuals. Older adults will develop closer relationships due to group activities. Synchronized goals, behaviors, and interests enable older adults to establish long-lasting connections in community-based elderly care, forming a virtuous cycle [14].

From the individual perspective of older adults, existing studies have analyzed the situation of older adults’ participation in rural community elderly care services. These studies mainly discuss the participation willingness, actual needs, and participation barriers of rural elderly people. A small number of studies have focused on how rural elderly people utilize different components of community elderly care services. This is a beneficial exploration for understanding the actual situation of older adults’ participation in rural community elderly care services. However, the fundamental purpose of developing rural community elderly care services is to enhance the well-being of older adults. In research on the impact of elderly care services on older adults’ well-being, the focus is mainly on the impact of community-level elderly care services on aspects such as older adults’ health level, health vulnerability, economic level, and participation in social activities.

In the academic discourse on the influence of elderly care services on the well-being of senior citizens, extant research has produced a series of significant findings. Shapiro et al. empirically demonstrated that the timely allocation of intervention services to elderly populations within underserved communities substantially elevates their subjective well-being, concomitantly reducing the risks of institutionalization and mortality [15]. Shinan-Altman et al.’s study of American seniors indicated that those situated in community-based care environments report heightened happiness levels and more optimistic outlooks regarding future health [16]. Drawing on a Chinese sample, Yang et al. established a robust positive correlation between community health services and the perceived meaning of life, as well as a sense of safety; notably, these services further fortify older adults’ confidence in their later-stage lives [17]. Hsieh et al. uncovered that home care services enable elderly individuals with chronic diseases to enhance their well-being in non-health domains, particularly in fostering friendships [18]. Despite these valuable contributions, a conspicuous gap persists in the current body of research: there is a dearth of in-depth investigations into the impact of elderly care services on subjective dimensions, such as life satisfaction, mental health status, and overall subjective well-being, among older adults. Moreover, there is an insufficient analysis of the mechanisms through which community elderly care services affect older adults’ well-being. Therefore, the impact of rural community elderly care services on older adults’ subjective well-being remains unclear. It is difficult to accurately evaluate the service effectiveness of current rural community elderly care services, and it is also difficult to guide future policy development.

Therefore, this study starts from the perspective of the subjective well-being of older adults. Centering on the core issue of the impact of rural community elderly care services on the subjective well-being of older adults, it further identifies the current development status of rural community elderly care services and empirically tests the impact of rural community elderly care services on the subjective well-being of older adults and its internal mechanisms. Thus, it provides empirical support for promoting the development of rural community elderly care services and enhancing the well-being level of older adults.

## 2. Conceptual Framework

Subjective well-being focuses on measurements from the psychological evaluation and subjective feelings of older adults, which is of great significance for enhancing older adults’ psychological satisfaction and value perception. It includes older adults’ life satisfaction, positive attitude towards life (positive emotions), and depression level (negative emotions). Therefore, this study mainly analyzes the impact of rural community elderly care services on the subjective well-being of older adults and its action mechanisms and examines whether rural elderly people can improve their subjective well-being levels by using four types of rural community elderly care services. This is to understand whether the current service contents truly meet older adults’ care needs and to provide a basis for government decision-makers to formulate more scientific and reasonable well-being policies and service plans for older adults.

The topic of subjective well-being has received increasing attention due to the influence of active aging. Previous studies based on theories related to social quality have examined the impact of different forms of social life mechanisms on the subjective well-being of older adults. For example, through social and economic security, social cohesion, social inclusion, and social empowerment, the subjective well-being of older adults can be substantially improved [19]. However, due to the ambiguity in the conceptual definition of subjective well-being, as it is related to aspects such as life satisfaction, emotional assessment, and self-esteem (and there are specific psychosocial goals to be achieved at different stages of life), existing studies generally use only one indicator, life satisfaction, to evaluate subjective well-being, thus showing insufficient attention to the subjective well-being of older adults [20]. In addition, with the development of society and the economy, basic living needs, such as housing and food, have been met, and people are seeking to satisfy higher-level needs related to lifestyle. However, as the most vulnerable group in society, older adults receive more attention in related social support research regarding their material life goals. It remains unclear how to fill the void in the social roles of older adults, especially rural elderly people [21].

**Figure 1:**
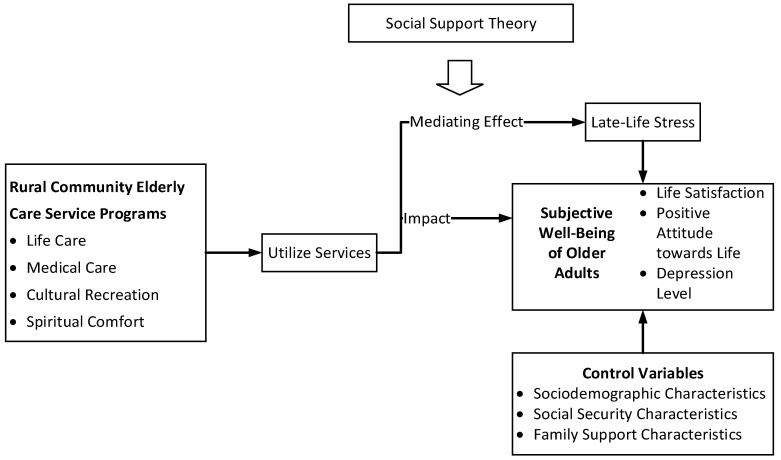
A framework for the impact of rural community elderly care services on the subjective well-being of older adults.

Based on the existing issues regarding the social support for older adults and their subjective well-being in the current research, this study constructs an analytical framework for the impact of rural community elderly care services on the subjective well-being of older adults, as shown in Figure 1, grounded in the social support theory. The social support theory contends that social support can substantially enhance an individual’s situation, mitigating the effects of stressors and adverse events—such as discrimination—on diverse outcomes, encompassing health, morbidity, mortality, and social and occupational adaptation [22]. A compelling illustration is found within life-course criminology, where ex-offenders typically confront severe hardships, including extreme poverty, financial insecurity, and housing inadequacies [23]. Through a reliance on material support from familial networks, friends, or governmental aid, these individuals can notably decrease their recidivism rates and facilitate their own successful social reintegration.

When applied to the context of aging, the social support theory elucidates the challenges faced by older adults. The aging process often precipitates a cascade of physiological, psychological, cognitive, economic, and social transformations, which may give rise to specific health risks, depressive symptoms, and feelings of social estrangement [24]. In response to the stressors encountered by older adults in their later life, scholars of social support have demonstrated that, despite the intensifying difficulties and pressures associated with aging, multifaceted support from various social actors can effectively alleviate stress and mitigate negative events, thereby enhancing the overall well-being of older adults [25].

This stress-buffering mechanism is activated when social support measures are tailored to address the needs triggered by stressful events, serving as a safeguard against potential detrimental effects [26]. In accordance with the social support theory, such support can not only improve the circumstances of older adults but also reduce the impact of stress and discrimination on various outcomes [27]. It enables them to re-establish their life purpose, alleviate symptoms of depression and anxiety, reinforce their sense of social value, and foster a positive psychological state.

Therefore, with the introduction of the concept of late-life stress, it has become evident that the utilization of community elderly care services by older adults can effectively alleviate such stress, thereby exerting a significant influence on their subjective well-being. This provides theoretical support for analyzing the impact of rural community elderly care services on the subjective well-being of older adults. The stress of later life plays a mediating role in the impact of community elderly care services on the subjective well-being of older adults. The following text further examines the impact of rural community elderly care services on the subjective well-being of older adults.

## 3. Methods

### 3.1. Data and Sample

This research is derived from a social survey titled ‘Development of Rural Elderly Care Services and the Living Conditions of Older Adults’ conducted by the author’s team from January to March 2021 in rural communities of three provinces in China, namely Shaanxi, Hebei, and Jiangsu. Given China’s vast territory and significant disparities in economic and social development among different regions, Jiangsu, Hebei, and Shaanxi provinces, which are typical in the development of rural community elderly care, were selected as the survey sites. In recent years, these three provinces have implemented policies to develop rural elderly care, ensuring the validity of this survey. The survey subjects were rural elderly people aged 60 and above. Only one elderly person was surveyed per household, and their consent was obtained prior to the survey. To ensure the quality of the survey, investigators were trained before the survey, with explanations given regarding potential problems during the survey. After the survey, team members reviewed the questionnaires.

This study adopted the sampling method of stratified random sampling. The plan was to select 900 rural elderly people and their villages as samples for a nationwide investigation in three layers. In the first layer, Shaanxi Province, Hebei Province, and Jiangsu Province were selected nationwide. In the second layer, approximately 8–10 counties were randomly selected within each province. In the third layer, 2–3 villages were randomly selected within each county. Finally, 10–15 rural elderly people were randomly sampled as survey subjects in each village. A total of 796 valid samples were obtained, yielding a response rate of 88.44%. A response rate exceeding 80% indicates the reliability of the sampling results, providing a robust foundation for subsequent analytical procedures.

### 3.2. Measures

(1) Dependent Variables

The dependent variable in this study is the subjective well-being of older adults. As described earlier, it is an assessment of the individual subjective perceptions of older adults, covering life satisfaction, positive life attitude, and level of depression.

In the measurement of life satisfaction, life satisfaction pertains to an individual’s comprehensive cognitive appraisal of their living circumstances. It is characterized by multi-dimensionality, complexity, and subjectivity, encompassing elements such as personal living standards, the sense of well-being, perceptions of the material foundation, economic conditions, and even the mode of elderly care. Previous relevant research commonly employs a subjective assessment of an individual’s life state, typically constructing it as either a four-category or five-category variable. The principle is that the higher the assigned value, the greater the life satisfaction, as elaborated in references [28,29]. Consequently, within this questionnaire, the item ‘Are you satisfied with your current life?’ is utilized to gauge the life satisfaction of older adults. The assigned values are defined as follows: 1 denotes ‘very dissatisfied’, 2 represents ‘somewhat dissatisfied’, 3 signifies ‘average’, 4 indicates ‘somewhat satisfied’, and 5 implies ‘very satisfied’. Evidently, the higher the score, the more content older adults are with their present lives.

In the measurement of older adults’ positive life attitude, a positive life attitude encompasses the optimistic, proactive, and self-actualizing mindsets that older adults adopt when confronting their later-life stages. Elderly individuals with a positive life attitude are more likely to cultivate and uphold positive outlooks on life and values. Previous research has identified two primary factors, namely a sense of happiness and hopefulness, to quantify the positive emotions experienced by older adults [30]. To further elucidate the connotation of the positive life attitude indicator, this study employs the item ‘Do you perceive your later-life as being happy and full of hope?’ within the questionnaire to assess the positive life attitude of older adults. The scoring system is defined as follows: 1 corresponds to ‘strongly disagree’, 2 represents ‘disagree somewhat’, 3 denotes ‘neutral’, 4 indicates ‘agree somewhat’, and 5 signifies ‘strongly agree’. Evidently, a higher score indicates that older adults hold a more positive attitude towards their current life.

When measuring the depression level of older adults, the globally acknowledged CESD-10 scale is employed to assess their depressive emotions [31]. The CESD-10 is a validated instrument designed for screening depressive symptoms, widely utilized in both community and clinical settings to identify and monitor depressive disorders, thereby aiding healthcare professionals in making informed diagnostic and treatment decisions. Comprising ten items, each item within this scale is scored on a scale from 0 to 3 points. Consequently, the cumulative score ranges between 0 and 30 points. Following standardization, it is important to note that a higher score corresponds to a lower depression level among older adults, indicating better mental health. Definitions and descriptive statistics of the subjective well-being variables are presented in Table 1 for a comprehensive understanding.

(2) Independent Variable

In this research, the independent variable is the utilization of rural community elderly care services by older adults. According to the service content, it is further classified into four categories: life care services, medical and nursing services, cultural and recreational services, and spiritual consolation services. For each service item, if an elderly individual does not utilize it, a value of 0 is assigned; conversely, if an older adult individual makes use of it, a value of 1 is assigned. Subsequently, the values of all service items are aggregated.

(3) Control Variables

Based on an examination of existing research, the control variables are classified into three dimensions: sociodemographic characteristics, social security characteristics, and family support characteristics. Sociodemographic characteristics encompass age, gender, marital status, educational level, family income, chronic illness status, disability status, smoking status, and alcohol-drinking status. Social security characteristics include medical insurance and pension insurance. Family support characteristics involve the number of children, the degree of children’s concern, and inter-generational support.

(4) Instrumental Variable

To tackle the endogeneity issue, this paper employs the approach of ‘analyzing the upper level’ for the selection of instrumental variables, as cited in references [32,33], and utilizes the ‘leave-one-out method’ [34]. Specifically, the average utilization intensity of community elderly care services within the respondent’s community, excluding the respondent themselves, is designated as the instrumental variable for the respondent’s utilization of community elderly care services. The rationality of this choice is founded on two key aspects. First and foremost, this instrumental variable exhibits a correlation with the variable of community elderly care service utilization, which functions as the independent variable in the model. Secondly, it demonstrates no correlation with the error term of the model. In terms of exogeneity, the average utilization intensity of elderly care services in the community, as investigated in this study, is derived by excluding the respondent and calculating the utilization proportion of all other elderly individuals within the community. Consequently, the utilization intensity of community elderly care services has no direct bearing on the respondent’s well-being level and other control variables, thereby satisfying the exogeneity principle of instrumental variables. Regarding correlation, owing to the presence of social network characteristics and peer effects within the community [35], the utilization intensity of elderly care services by other community members exerts an influence on the respondent’s decision-making process regarding participation in community elderly care services. This meets the requirement of a direct correlation between the instrumental variable and the explanatory variable. Therefore, the selection of this instrumental variable is deemed reasonable.

(5) Mediating Variable

Regarding the measurement of late-life stress, this study draws on relevant research and conducts an investigation from three aspects: older adults’ health-related stress, economic stress, and living-environment-related stress [36,37]. Respondents are asked to evaluate their stress levels in these three aspects. Each question is scored on a scale from 1 to 5, with a total of 15 points. A higher score indicates that older adults experience less stress.

### 3.3. Statistical Analysis

In this study, regression analysis techniques are meticulously employed to empirically validate the influence of rural community elderly care services on the subjective well-being of older adults. Initially, an examination of the relationship between rural community elderly care services and the subjective well-being of older adults is carried out. When the dependent variable assumes a continuous nature, the ordinary least squares (OLS) multiple linear regression approach is applied. Conversely, when the dependent variable is an ordered variable, the ordered probit (Oprobit) regression model is utilized, accompanied by the calculation of its marginal effect outcomes.

To safeguard the robustness of the research findings and circumvent potential endogeneity concerns, such as omitted variables and reverse causality, the two-stage least squares model with the incorporation of instrumental variables (IV-2SLS) is further implemented for regression analysis. This step serves to reaffirm the stability and reliability of the results obtained. Instrumental variables are employed in disciplines such as economics, social sciences, and medical research to address endogeneity challenges in causal inference.

Regarding the mechanism analysis, grounded in the theoretical interpretation of social support theory within the established analytical framework, the mediating effect model is deployed to conduct a comprehensive exploration of the underlying mechanisms. This enables a more profound understanding of the causal pathways through which rural community elderly care services impact the subjective well-being of older adults. Using regression analysis, the following sequential tests are conducted: first, the total effect of the independent variable on the dependent variable is examined; second, the effect of the independent variable on the mediating variable is assessed; and finally, the combined effects of the independent and mediating variables on the dependent variable are tested to determine whether a mediating effect exists.

## 4. Results

### 4.1. Rural Community Elderly Care Services and Subjective Well-Being of Elderly People

Table 2 reports the impact of older adults’ utilization of rural community elderly care services on their subjective well-being. Among them, Model 1 for life satisfaction and Model 2 for positive life attitude adopt the Oprobit model, while Model 3 for depressive level adopts the OLS model.

Based on the regression outcomes presented in Table 2, it is evident that rural community elderly care services exert a significantly positive influence on the life satisfaction, positive life attitude, and depressive level within the subjective well-being framework of older adults, all at the 1% significance level.

In the context of life satisfaction, rural community elderly care is capable of delivering multifaceted support services, covering areas like caregiving, medical services, and social engagement, which substantially elevates the quality of life for older adults. This comprehensive support system enables them to lead a more comfortable and fulfilling life in their later years.

Concerning the positive life attitude, the personalized services offered by rural community elderly care across multiple dimensions play a crucial role in catering to the diverse requirements of older adults. By doing so, it not only enhances their autonomy in later life but also fortifies their positive life outlook. This empowerment allows older adults to actively participate in their daily lives and maintain a positive mindset.

Regarding the depressive level, rural community elderly care services establish a robust social support network for older adults. Through attentive care and continuous support, these services effectively mitigate the negative emotions experienced by older adults, thereby making a substantial contribution to the amelioration of their emotional and mental conditions. This support network serves as a buffer against feelings of isolation and depression, promoting better mental health among older adults.

In terms of regression coefficients, the parameter value denoting the impact of rural community elderly care services on the depressive level of the rural elderly population is 1.438. This implies that, under the condition of all the other factors remaining constant, a one-unit increment in the utilization of rural community elderly care services can lead to a 1.438-unit improvement in the depressive state of rural elderly individuals, indicating a reduction in their depressive level.

The parameter estimates derived from the Oprobit model merely offer insights into the direction and statistical significance of how the utilization of rural community elderly care services impacts the subjective well-being of older adults. However, the practical implications of the coefficient values are not straightforwardly comprehensible. Consequently, to gain a more in-depth understanding, the marginal effects of the utilization of rural community elderly care services on life satisfaction and a positive life attitude were further computed, and the findings are presented in Table 3.

When all explanatory variables are set at their mean values, for every one-unit increase in the utilization of rural community elderly care services, the probabilities of rural elderly people reporting ‘very dissatisfied’, ‘fairly dissatisfied’, and ‘average’ levels of life satisfaction decline by 1.1%, 3.0%, and 6.5%, respectively. In contrast, the probabilities of ‘fairly satisfied’ and ‘very satisfied’ increase by 1.0% and 9.7%, respectively.

Analogously, for each one-unit increase in the utilization of rural community elderly care services, the probabilities of rural elderly people expressing ‘strongly disagree’, ‘disagree somewhat’, and ‘neutral’ regarding their positive life attitude decrease by 1.3%, 4.3%, and 1.6%, respectively. Meanwhile, the probabilities of ‘agree somewhat’ and ‘strongly agree’ increase by 1.5% and 5.7%, respectively.

### 4.2. Sensitivity Analysis

Table 4 presents the IV-2SLS estimation results regarding the influence of rural community elderly care services on the subjective well-being of older adults. Notably, both the significance level and the direction of the impact are in line with the findings reported earlier in this paper. This consistency strongly suggests that the outcomes of the benchmark regression are robust, thereby validating the reliability and stability of the previously obtained results within the context of this research.

### 4.3. Mediation Analysis

As elaborated in the previous discussion on social support theory, once social support measures respond to the demands triggered by stress-inducing events, a stress-buffering mechanism is activated. This mechanism serves to counteract the adverse effects stemming from stress. Consequently, social support can effectively enhance an individual’s state, reducing the impact of stress and negative incidents, such as discrimination, on a wide range of outcomes. Specifically, it can assist older adults in rediscovering their life objectives, alleviating symptoms of depression and anxiety. Moreover, it helps to strengthen their sense of social value and maintain a positive mental attitude. In light of this, based on ‘late-life stress’ as a mechanism variable, this research further explores whether it exerts a mediating effect in the process of how rural community elderly care services influence the subjective well-being of older adults.

The results are presented in Table 5. In Model 1, the estimated coefficient of the utilization of rural community elderly care services is 0.423, and it is statistically significant at the 1% level. This finding strongly supports the hypothesis that rural community elderly care services can effectively reduce the life stress experienced by older adults.

Moreover, in Model 2, Model 3, and Model 4, both rural community elderly care services and late-life stress exhibit significant positive correlations at the 1% level. When combined with the previously established positive correlations between rural community elderly care services and older adults’ life satisfaction, positive life attitude, and depression levels, it provides robust evidence for the existence of a mediating effect. This set of results convincingly demonstrates that rural elderly individuals can mitigate the negative impacts of late-life stress by accessing community elderly care services. As a result, their overall level of subjective well-being is notably enhanced.

## 5. Discussion

This paper, by introducing the social support theory, constructs an analytical framework for exploring the impact of rural community elderly care services on the subjective well-being of older adults. Employing a comprehensive approach that incorporates multiple linear regression, ordered probit regression, the IV-2SLS model, and the mediating effect model, it conducts an in-depth examination of whether rural elderly individuals can elevate their subjective well-being through the utilization of rural community elderly care services. Simultaneously, this paper delves into the underlying mechanisms to gain insights into whether the current service content genuinely caters to older adults’ endowment needs.

The results of this study demonstrate that rural community elderly care services can effectively enhance life satisfaction, foster a positive life attitude, and ameliorate the depressive level within the subjective well-being framework of older adults. The underlying mechanism is as follows: When older adults utilize rural community elderly care services, the social support embedded in these services can significantly improve their individual situations. This improvement helps buffer the negative impacts exerted by stressors and adverse events, like discrimination, on various life aspects. For example, it helps older adults re-establish their life goals, alleviates symptoms of depression and anxiety, and strengthens their sense of social value, thus enabling them to maintain a positive psychological state.

Previous research has established that older adult care services provided by communities play a pivotal role in elevating the quality of life for older adults [38]. Moreover, for older adults with care requirements, an augmentation in the quantity and accessibility of community services has been shown to significantly improve their living conditions [39]. Evidently, rural community elderly care services, as a fundamental component of grass-roots elderly care services, can effectively alleviate older adults’ daily life-related care burdens in terms of service efficacy and concurrently enhance their quality of life. This study, delving deeper from the perspective of subjective well-being, reveals that the utilization of rural community elderly care services can assist older adults in cultivating a more positive life attitude and remarkably reduce their depressive levels by improving their living situations.

Relevant studies posit that the enhancement of the mental health of rural elderly individuals at the community level primarily emanates from hardware-related factors, including the locational and transportation environment, the basic living environment, and an older-adult-friendly facility environment [40,41]. The findings of this study suggest that within the rural community elderly care model, through the provision of medical and psychological support to older adults as well as the creation of more opportunities for social interaction in cultural and recreational activities, the stress and anxiety experienced by older adults can be effectively mitigated, thus promoting the development of their mental health.

Existing research on the emotional characteristics of older adults has primarily focused on the measurement of dimensions such as depression, anxiety, and loneliness. By contrast, this study shifts its focus to the positive emotional experiences of older adults, demonstrating that an engagement with rural community elderly care services helps cultivate a more optimistic and proactive life attitude by improving their late-life living conditions. While prior studies have extensively explored the policy impacts of community-based care services [17], they have placed a disproportionate emphasis on assessing direct effects, leaving the analysis of underlying causal mechanisms underdeveloped.

Grounding itself in social support theory, this study constructs a theoretical analytical framework to explicatively model how rural older adults’ participation in community care services influences their subjective well-being. A quantitative methodology is employed to empirically investigate the intricate mediating pathways through which these services affect well-being. This research yields two distinct contributions. First, from the perspective of direct beneficiaries, it uncovers rural older adults’ policy perceptions toward community care services, providing empirical evidence to inform evidence-based evaluations of policy effectiveness for governmental agencies. Second, from a policy-making standpoint, it offers a nuanced understanding of the impact mechanisms through which rural community care services shape the well-being of older adults. This understanding enables policymakers to design targeted interventions aimed at enhancing participation in rural community care services and fostering holistic well-being outcomes. By bridging micro-level individual experiences with macro-level policy implications, this study enriches both the theoretical discourse and practical applications in the field of rural elderly care.

However, as an effective exploration to address rural elderly care challenges, the developmental patterns of rural community elderly care services differ across stages. Cross-sectional survey data can only reflect conditions at a specific time point, thus failing to analyze temporal changes. Therefore, future research should conduct long-term observations of these services and use longitudinal data to establish conclusions with a broad applicability and validity.

## 6. Implications and Limitations

### 6.1. Implications

Rural community elderly care services play a pivotal role as a crucial means of refining the rural elderly care service system. The overarching objective is to augment the well-being of the rural elderly population. This study diverges from previous research endeavors that were primarily centered on aspects such as the demand for rural community elderly care services, providers’ willingness to supply, and the level of elderly participation. Instead, it zeroes in on the ‘obligation’ of elderly care services to effectively enhance the well-being of older adults. By doing so, it not only broadens the scope of the research on rural elderly care services but also perfects the research logic that links the utilization of services to the elevation of well-being. Moreover, it enriches the theoretical exploration of elderly care policies within the practical context of China.

At the theoretical level, this study adopts a welfare-oriented viewpoint. It specifically delves into the extent to which the existing rural community elderly care services can contribute to the enhancement of the subjective well-being of rural seniors. Moreover, it elaborates on the internal mechanisms through which these services influence the well-being of older adults. This research establishes a comprehensive theoretical analytical framework for exploring the development of rural community elderly care. In the process, it expands the application scope of welfare economics in the field of elderly care services, providing valuable insights for rural public governance. By doing so, it not only enriches the theoretical basis of rural elderly care research but also offers practical guidance for the improvement and development of rural community elderly care services.

At the practical level, this research provides decision-making support for improving rural community elderly care policies. It comprehensively analyzes rural community elderly care services, identifies their impact on the subjective well-being of the rural elderly, and explores how service utilization enhances well-being. From the perspective of the rural elderly, the direct beneficiaries, this study reveals their understanding of community elderly care policies, offering evidence for government departments to evaluate policy effectiveness. From the government’s perspective, it helps them understand the impact mechanisms of these services, enabling them to explore strategies to boost service participation and improve older adults’ well-being. In conclusion, this research identifies key areas for ensuring the sustainable development of rural community elderly care services and strengthening the rural elderly care service system, which can guide future policy-making and implementation.

### 6.2. Limitations and Future Research

This research utilizes cross-sectional survey data to disclose the present developmental state of rural community elderly care services from a welfare-oriented perspective. By doing so, it comprehensively exposes the divergences among samples, thereby enabling a comprehensive understanding of how current rural community elderly care services contribute to the enhancement of older adults’ well-being. Nevertheless, rural community elderly care services, being an effective attempt to overcome the rural elderly care predicament, exhibit distinct developmental patterns at various stages. The utilization of cross-sectional survey data is confined to reflecting the development scenario at a specific moment and fails to analyze the alterations across the temporal dimension. Consequently, in the future, it is imperative to carry out long-term observations of rural community elderly care services. Moreover, longitudinal data should be harnessed to derive conclusions that possess a broad applicability and validity, which will in turn strengthen the research foundation in this field.

## 7. Conclusions

Grounding itself in the social support theory, this study integrates the practical circumstances of rural elderly care service development in China to establish a theoretical analytical framework. This framework is designed to explore the influence of rural seniors’ participation in community-based elderly care services on their subjective well-being. Under the guidance of this theoretical framework, the subjective well-being of older adults is deconstructed and reconstructed from three distinct dimensions: life satisfaction, a positive life attitude, and the level of depression. Through rigorous examination, the impact of rural community elderly care services on the subjective well-being of older adults is investigated.

The results reveal that rural community elderly care services can boost the subjective well-being of older adults by mitigating the life pressures they encounter in their later years. The coefficient for the effect of rural community elderly care services on depressive levels was 1.438, suggesting that a one-unit increase in the utilization of these services reduces depressive levels by 1.438 units, holding all other variables constant. Regarding life satisfaction and a positive life attitude, when all explanatory variables are set at their mean values, for every one-unit increase in the utilization of rural community elderly care services, the probabilities of rural elderly reporting ‘very dissatisfied’, ‘fairly dissatisfied’, and ‘average’ levels of life satisfaction decline by 1.1%, 3.0%, and 6.5%, respectively. In contrast, the probabilities of ‘fairly satisfied’ and ‘very satisfied’ increase by 1.0% and 9.7%, respectively. Analogously, for each one-unit increase in the utilization of rural community elderly care services, the probabilities of rural elderly selecting ‘strongly disagree’, ‘disagree somewhat’, and ‘neutral’ regarding their positive life attitude decrease by 1.3%, 4.3%, and 1.6%, respectively. Meanwhile, the probabilities of ‘agree somewhat’ and ‘strongly agree’ increase by 1.5% and 5.7%, respectively. Consequently, focusing on the adverse effects of the aging process in aspects such as physical health, mental state, cognitive ability, economic situation, and social status, which older adults face during their later-life stages, stands as the linchpin for enhancing their well-being.

Ultimately, these research findings hold substantial reference value for formulating practical strategies for community elderly care services that align with the genuine needs of older adults. This involves strengthening the demand-driven supply of rural community elderly care services. In terms of service offerings, customized elderly care service plans should be crafted in accordance with older adults’ health conditions, personal interests, and individual requirements, thereby satisfying their diverse and personalized elderly care service needs.

In international practice, despite cross-national variations in rural social systems and family structures, the logic of how community services address the emotional and functional needs of older adults carries cross-cultural applicability, offering valuable insights for rural elderly care policies in developing countries and across the globe. It is recommended that international practices focus on the common pathway of ‘community services–social support–well-being enhancement’, integrating local cultural and institutional contexts to optimize service models and thereby promote the improvement of well-being for rural elderly populations in various countries.

## Figures and Tables

**Table 1:** Definitions and descriptive statistics of subjective well-being variables.

Variable	Variable Definition	Mean	Std. Dev.
Life Satisfaction	1 = very dissatisfied, 2 = somewhat dissatisfied, 3 = average, 4 = somewhat satisfied, 5 = very satisfied	3.87	0.90
Positive Life Attitude	1 = strongly disagree, 2 = disagree somewhat, 3 = neutral, 4 = agree somewhat, 5 = strongly agree	3.60	1.13
Depressive Level	Measured by the CESD-10 self-rating depression scale: ranging from 0 to 30 points	20.90	6.52

**Table 2:** The regression analysis of rural community elderly care services on the subjective well-being of older adults.

Variable	Oprobit	Oprobit	OLS
	Model 1	Model 2	Model 3
	Life Satisfaction	Positive Life Attitude	Depressive Level
Rural Community Elderly Care	0.335 ***	0.203 ***	1.438 ***
Services	(0.041)	(0.039)	(0.202)
Age	0.015 **	0.003	−0.002
	(0.007)	(0.007)	(0.039)
Gender	0.138	0.024	0.985 *
	(0.096)	(0.091)	(0.518)
Marital Status	0.117	0.152	1.145 *
	(0.105)	(0.094)	(0.593)
Educational Level	−0.024	0.153 ***	0.422
	(0.059)	(0.055)	(0.330)
Family Income	0.000 ***	0.000	0.000
	(0.000)	(0.000)	(0.000)
Chronic Illness Status	−0.453 ***	−0.359 ***	−1.865 ***
	(0.096)	(0.095)	(0.482)
Disability Status	−0.097 *	−0.160 ***	−1.278 ***
	(0.050)	(0.048)	(0.293)
Smoking Status	−0.042	−0.099	−0.028
	(0.105)	(0.098)	(0.542)
Alcohol-Drinking Status	0.019	0.068	0.567
	(0.095)	(0.092)	(0.498)
Medical Insurance	0.041	−0.091	−0.481
	(0.129)	(0.129)	(0.662)
Pension Insurance	−0.063	0.010	1.390 **
	(0.096)	(0.101)	(0.545)
Number of Children	0.013	0.012	−0.145
	(0.034)	(0.034)	(0.209)
Degree of Children’s Concern	0.043	0.155 ***	0.394 *
	(0.038)	(0.036)	(0.214)
Inter-Generational Support	−0.000	0.000	0.000 *
	(0.000)	(0.000)	(0.000)
Cons			16.611 ***
			(3.091)
*N*	796	796	796
R-squared			0.175
pseudo R^2^	0.063	0.053	

Robust standard errors in parentheses. * *p* < 0.10, ** *p* < 0.05, and *** *p* < 0.01.

**Table 3:** Marginal effects of rural community elderly care services on life satisfaction and positive life attitude.

Model 1	Rural Community	Model 2	Rural Community
Life Satisfaction	Elderly Care Services	Positive Life Attitude	Elderly Care Services
Very Dissatisfied	−0.011 ***	Strongly Disagree	−0.013 ***
	(0.003)		(0.003)
Fairly Dissatisfied	−0.030 ***	Disagree Somewhat	−0.043 ***
	(0.005)		(0.008)
Average	−0.065 ***	Neutral	−0.016 ***
	(0.008)		(0.003)
Fairly Satisfied	0.010 **	Agree Somewhat	0.015 ***
	(0.005)		(0.004)
Very Satisfied	0.097 ***	Strongly Agree	0.057 ***
	(0.011)		(0.011)
Control Variables	YES	Control Variables	YES
*N*	796	*N*	796

Robust standard errors in parentheses. ** *p* < 0.05, and *** *p* < 0.01.

**Table 4:** IV-2SLS estimation of impact of rural community elderly care services on subjective well-being of older adults.

Equation Specification	(1)	(2)	(3)	(4)
	The First Stage	The Second Stage		
Dependent Variables	Rural Community Elderly Care Services	Life Satisfaction	Positive Life Attitude	Depressive Level
Rural Community Elderly		0.245 ***	0.225 ***	1.820 ***
Care Services		(0.039)	(0.049)	(0.277)
Instrumental Variable	0.948 ***			
	(0.030)			
Control Variables	YES	YES	YES	YES
Cons	−0.157	2.796 ***	2.572 ***	16.268 ***
	(0.337)	(0.409)	(0.512)	(2.904)
*N*	796	796	796	796

Robust standard errors in parentheses. *** *p* < 0.01.

**Table 5:** Mediating effect of rural community elderly care services on the subjective well-being of older adults.

	Model 1	Model 2	Model 3	Model 4
Variable	Late-Life Stress	Life Satisfaction	Positive Life Attitude	Depressive Level
Rural Community Elderly	0.423 ***	0.215 ***	0.119 ***	1.004 ***
Care Services	(0.093)	(0.030)	(0.035)	(0.185)
Late-life Stress		0.087 ***	0.161 ***	1.028 ***
		(0.012)	(0.013)	(0.075)
Age	0.033**	0.007	−0.004	−0.036
	(0.017)	(0.005)	(0.006)	(0.034)
Gender	−0.120	0.114	0.020	1.109 **
	(0.234)	(0.071)	(0.081)	(0.459)
Marital Status	0.368	0.058	0.089	0.767
	(0.234)	(0.077)	(0.086)	(0.511)
Educational Level	0.571 ***	−0.077 *	0.055	−0.165
	(0.132)	(0.046)	(0.048)	(0.320)
Family Income	0.000 *	0.000 ***	0.000	0.000
	(0.000)	(0.000)	(0.000)	(0.000)
Chronic Illness Status	−1.098 ***	−0.230 ***	−0.137 *	−0.736 *
	(0.231)	(0.068)	(0.078)	(0.443)
Disability Status	−0.449 ***	−0.039	−0.092 **	−0.817 ***
	(0.116)	(0.040)	(0.044)	(0.237)
Smoking Status	−0.182	−0.019	−0.065	0.159
	(0.239)	(0.078)	(0.091)	(0.513)
Alcohol-Drinking Status	0.204	−0.003	0.063	0.357
	(0.229)	(0.070)	(0.082)	(0.450)
Medical Insurance	−0.161	0.049	−0.030	−0.315
	(0.307)	(0.092)	(0.115)	(0.563)
Pension Insurance	−0.097	−0.025	0.044	1.490 ***
	(0.228)	(0.072)	(0.093)	(0.475)
Number of Children	−0.174 **	0.030	0.038	0.034
	(0.087)	(0.025)	(0.030)	(0.181)
Degree of Children’s Concern	0.239 ***	0.010	0.116 ***	0.149
	(0.086)	(0.028)	(0.032)	(0.189)
Inter-Generational Support	−0.000	−0.000	0.000 **	0.000 ***
	(0.000)	(0.000)	(0.000)	(0.000)
Cons	7.202 ***	2.166 ***	1.447 ***	9.207 ***
	(1.292)	(0.411)	(0.484)	(2.743)
*N*	796	796	796	796
R-squared	0.151	0.203	0.271	0.340

Robust standard errors in parentheses. * *p* < 0.10, ** *p* < 0.05, and *** *p* < 0.01.

## Data Availability

The data presented in this study are available from the corresponding author upon request.
